# Hydrophilic inflatable penile prosthesis surface coatings readily rebind antibiotics and maintain antimicrobial efficacy ex vivo

**DOI:** 10.1038/s41443-025-01104-8

**Published:** 2025-06-11

**Authors:** Brian H. Im, Aaron R. Hochberg, Analyse H. Giordano, Rachel Evans, Andrzej Fertala, Jolanta Fertala, Noreen J. Hickok, Paul H. Chung

**Affiliations:** 1 Department of Urology, Sidney Kimmel Medical College, Thomas Jefferson University, Philadelphia, PA USA; 2 Department of Orthopaedic Surgery, Sidney Kimmel Medical College, Thomas Jefferson University, Philadelphia, PA USA

**Keywords:** Translational research, Drug delivery

## Abstract

Hydrophilic penile prosthesis (PP) surfaces removed during revision surgery may potentially rebind antiseptics and maintain antimicrobial efficacy ex vivo. Coloplast Titan reservoirs and cylinders were retrieved during revision surgery for mechanical failure. Congo Red staining and contact angle measurements were performed to evaluate the integrity of the hydrophilic surface. Fluorescent antibiotic binding was performed by submerging discs for 3 min in either fluorescein-isothiocyanate (FITC) labeled or unlabeled vancomycin at 2 mg/mL. Fluorescence was quantified via ImageJ. 8 mm discs were submerged for 3 min in normal saline (NS), 0.05% chlorhexidine gluconate, or a 2 mg/mL vancomycin and 160 μg/mL gentamicin (VG) antibiotic, then incubated with 10^5^ colony-forming units per milliliter of methicillin-sensitive *Staphylococcus aureus* ATCC25923 for 48 h then counted. Mann-Whitney U and one-way ANOVA tests were performed to compare outcomes, p < 0.05 considered significant. All PP tested for binding with FITC-vancomycin showed significant increases in fluorescence relative to unlabeled vancomycin controls (p < 0.0001). All explanted and control PP exhibited significant decreases in bacterial counts on VG-treated surfaces relative to NS controls (p < 0.01). The hydrophilic surface of the reservoir and cylinders maintain their integrity based on Congo Red staining and rebind VG antibiotics effectively based on microbiology and fluorescent binding studies.

## Introduction

Penile prosthesis (PP) surgery generally has excellent outcomes and results in high levels of patient satisfaction; however, revision surgery is required in 7–8% of cases [1–3]. With revision surgery, complication risk relative to primary PP surgery increases, most notably a significantly higher risk for infection [4, 5]. While primary PP surgery has an infection rate of around 1–2%, revision surgery has a reported infection rate of nearly 10% [4, 6]. As such, adequate infection control is critical in the setting of PP revision surgery.

During revision PP surgery, existing PP components may be retained rather than replaced with a new component (i.e., for repair of an impending erosion, reservoir herniation, or pump relocation) [7]. Using a hydrophilic, commercially-available PP surface, prior studies from our laboratory demonstrated potential rebinding properties of this hydrophilic surface using new PPs in vitro. We demonstrated that following a 0.05% chlorhexidine gluconate (CHG) dip, with immediate irrigation again with CHG, the surface lost all antimicrobial activity. In contrast, if this CHG-dipped surface was irrigated with a vancomycin and gentamycin (VG) solution, the PP exhibited antimicrobial properties on the PP [8]. We thus suggested that VG was able to bind to the hydrophilic surface as part of the irrigation, even when it had been previously dipped in 0.05% CHG.

Based on our previous studies of new surfaces, we asked if these retained hydrophilic PP surfaces can rebind antiseptic agents during revision surgery to confer antimicrobial protection. In this manuscript, we evaluate the potential antimicrobial rebinding properties of explanted hydrophilic PP surfaces.

## Methods

### Penile prosthesis material, explant sterilization, and congo red staining

The hydrophilic PPs were retrieved from medically-necessary revision surgeries. These PPs would normally be discarded and had no patients’ identifiers so that they were classified as “not human research” and IRB approval was not required for this study. Coloplast Titan® reservoirs (n = 5) and cylinders (n = 2) (Coloplast®, Minneapolis, MN) were sourced from 5 patients who underwent revision surgery for mechanical malfunction. Congo Red staining of the PP was initially performed in the operating room to assess the integrity of the hydrophilic surface immediately after being removed from the patient [9]. A Congo Red staining solution at 25 g/L was made using sterile water according to manufacturer recommendations (ThermoFischer Scientific™, Waltham, MA). PP were submerged in Congo Red solution for 5 min, after which the material was rinsed in sterile NS to remove any loosely adherent dye, covered in aluminum foil to protect from light which could cause degradation of the Congo Red dye, dried in a laminar flow hood for two hours, and evaluated at low and high magnification microscopically, using a Nikon® Eclipse E600 microscope (Tokyo, Japan) [10].

To reduce risk of handling patient specimens in the laboratory and remove any pre-existing biofilm and intra-operative bacterial contamination which may have affected study results, PPs were cleaned via sonication in sterile normal saline (NS) for 20 min, followed by multiple rinses in NS, and finally submersion in 70% EtOH for 30 min [11, 12]. These washed PPs were then covered with sterile towels and dried in a laminar flow hood for 2 h, after which they were stored in sterile petri dishes for a minimum of 24 h prior to use. Recovered PPs were then stained with Congo Red in the lab to evaluate the integrity of the hydrophilic surface after cleaning. To assess any effects of the sterilization and cleaning protocols, we stained both new and ex vivo PPs with the same 25 g/L Congo Red solution after each step of the PP sterilization process to assess if the cleaning process alters the hydrophilic surface, and evaluated them both grossly and microscopically [9].

A new, sterile PP reservoir bearing the hydrophilic coating was obtained from the manufacturer and used as the control. Discs were cut from the reservoirs in sterile fashion using 8 mm diameter biopsy punches (Sklar®, West Chester, PA).

### Contact angle measurement

In order to further confirm that the sterilization process has not affected the hydrophilicity of the PP surface, contact angles were measured on PP hydrophilic surfaces using the sessile drop technique. Contact angles are the angles made at the interface of a liquid and a solid surface, and are measured to quantify the wettability of the surface by a liquid, a function of the hydrophilicity and/or hydrophobicity of the surface. We can use the contact angle measurements to ensure that the sterilization and cleaning processes do not alter the hydrophilicity of the surface [13]. 6 mm PP discs sourced from reservoirs, underwent either no sterilization, sonication, or sonication with a 70% EtOH soak, followed by drying for 2 h. Dried discs were glued to microscope slides with the hydrophilic-coated side facing up, after which a level plane was established. 10 μL of deionized water was slowly pipetted directly onto each of the PP discs, after which perpendicular views of each droplet were photographed. Contact angles were directly measured between a tangential line and the PP disc surface [14].

### Evaluation of hydrophilic surface rebinding using fluorescein isothiocyanate (FITC) labeled vancomycin

Sterilized and dried PP discs from both new and explanted reservoirs were viewed under a fluorescent microscope to assess the baseline auto-fluorescence of the PP. To then visually assess the rebinding of the antibiotic to the PP hydrophilic surfaces, fluorescein-isothiocyanate (FITC)-labelled vancomycin (2 mg/mL in NS; Sigma Aldrich®, St. Louis, MO) (VAN-FITC) or unlabeled vancomycin (2 mg/mL in NS) controls were incubated with the discs for 3 min, followed by rinsing in NS to remove unbound antibiotic. Non-labeled vancomycin was used as a control group, in order to account for any autofluorescence that non-labeled vancomycin and the hydrophilic surface itself may have.

PPs were imaged using a Nikon Eclipse® E600 microscope (λex = 500 nm). Fluorescent microscopy images were analyzed in ImageJ® (NIH, Bethesda, MD), where mean gray values were determined to calculate fluorescence intensity.

### Evaluation of antimicrobial activity of explanted hydrophilic surfaces

**Fig. 1:**
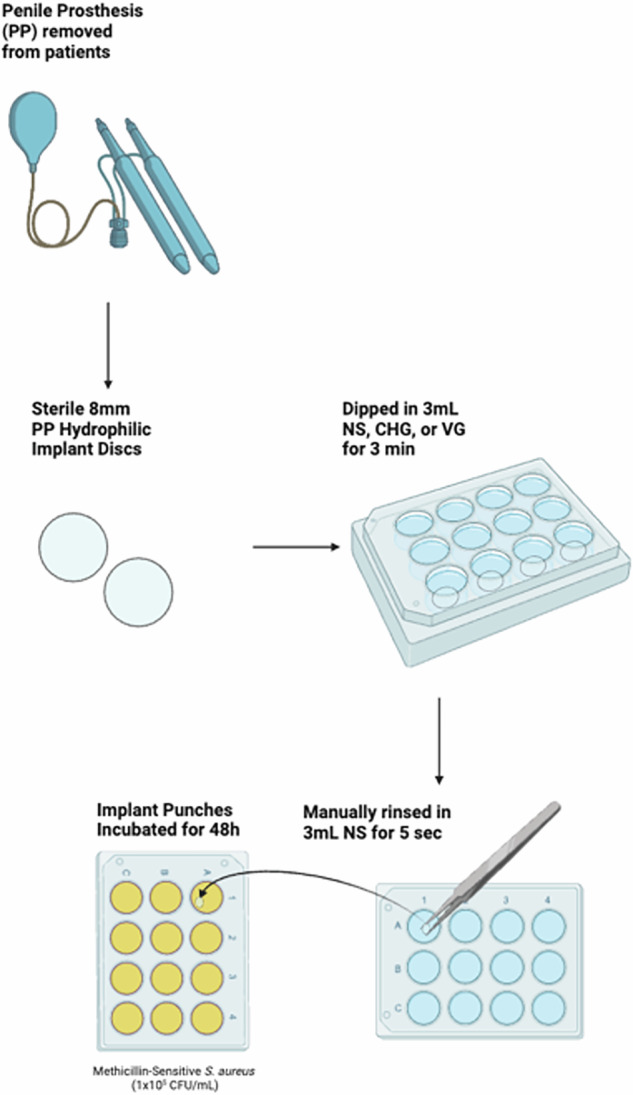
Antiseptic Rebinding Bacterial Assay Protocol. NS normal saline, VG vancomycin + gentamicin, CHG chlorhexidine gluconate.

Explanted, washed, sterilized, and dried PP hydrophilic surfaces from both new reservoirs and each of the explanted PP reservoirs and cylinders were tested for their ability to re-bind antimicrobial solutions, as evidenced by decreased bacterial colonization. Vancomycin and gentamicin were constituted to a stock solution of 10 mg/mL and 800 μg/mL respectively in 50 mL of NS, after which a 1:5 dilution was made to achieve a 2 mg/mL vancomycin and 160 μg/mL gentamycin solution. 8 mm PP discs (n = 12) were dipped in NS, 0.05% CHG, or the 2 mg/mL vancomycin and 160 μg/mL gentamycin (Sigma-Aldrich®, Burlington, MA) solution (VG), then rinsed in 2 mL of NS to remove any excess solution. The treated discs were then incubated in Methicillin-sensitive *Staphylococcus aureus* (MSSA) solution for 48 h, after which adherent surface bacteria were suspended, diluted, plated, and counted (Fig. 1).

### Bacterial strain, incubation, and recovery of adherent bacteria

Methicillin-sensitive *Staphylococcus aureus* (MSSA) ATCC®25923^™^ (ATCC®, Manassas, VA) was cultured overnight in tryptic soy broth (TSB, BD Biosciences®, Franklin Lakes, NJ) and diluted with phosphate-buffered saline (PBS, MP Biomedicals®, Irvine, CA) using turbidity to achieve 1×10^7^ colony forming units (CFU)/mL. 0.5 mL of the 1 × 10^7^ bacterial solution was added to 49.5 mL of TSB; this 1:100 dilution achieved a final bacterial incubation solution of 1 × 10^5^ CFU/mL (containing <0.1% PBS) [14, 15]. The treated discs were incubated at 37 °C under static conditions for 48 h in 12-well plates containing 3 mL of our bacterial incubation solution.

After incubation (Fig. 1), PP discs were harvested by gently rinsing with PBS to remove loosely bound bacteria from the disc surface, followed by incubating the PP discs with a 0.3% Tween-20 solution for 30 min at 37 °C in a shaker set at 180 RPM to suspend the adherent bacteria. The resulting suspended bacteria were serially diluted, plated onto PetriFilms™ (3M®, Maplewood, MN), and counted.

### Statistical analysis

All statistical testing was performed using Prism 7™ (GraphPad®, San Diego, CA). Mann-Whitney U and one-way ANOVA tests were performed to compare outcomes, p < 0.05 considered significant.

## Results

### Congo-Red staining to evaluate the integrity of the hydrophilic surface

**Fig. 2:**
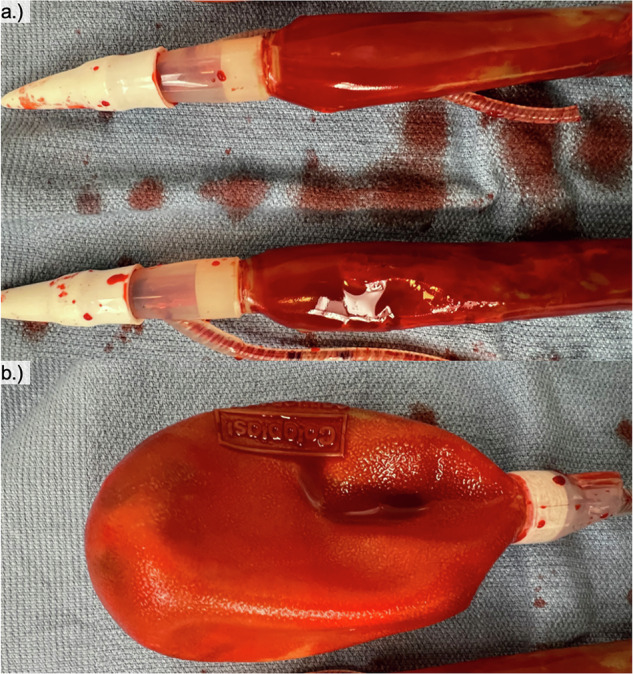
Unsterilized, removed implant components stained with Congo Red in the operating room. **a** Implant cylinders stained with Congo Red. **b** Implant reservoir stained with Congo Red.

Immediately after removal in the operating room, cylinders and a reservoir were stained with Congo Red (Fig. 2). Macroscopically, staining patterns of the native implant, Explant #4 and Explant #5 cylinders and reservoirs were similar (data not shown). However, the rear-tip extenders, proximal PP cylinder tip, and the reservoir lock-out valve showed no staining. Microscopically, there existed heterogeneity in staining patterns, with some portions of the PP cylinders and reservoirs adsorbing less of the Congo Red stain per field of view.

**Fig. 3:**
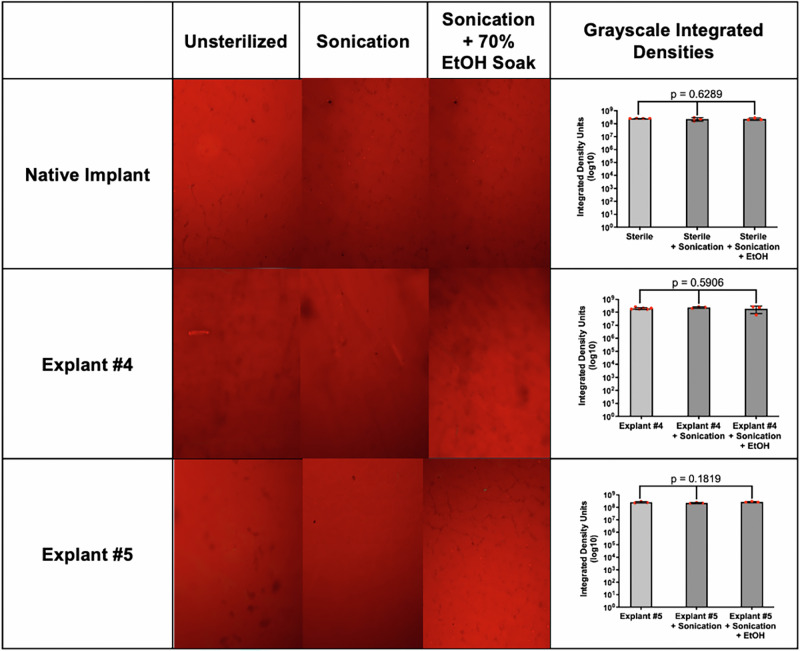
Native PPs and retrieved ex vivo reservoirs were stained with Congo Red as-is, following sonication protocol, and following sonication + EtOH sterilization protocol. Congo Red staining quantified by grayscale integrated densities and plotted along with 95% with standard deviations, n = 3 for each condition. Statistical significance was determined by one-way ANOVA.

We then examined the PP surface after retrieval and after each step of sterilization (Fig. 3). All PPs throughout the figure showed bright red and darker areas of staining, independent of source or treatment. The unsterilized PPs appeared similar, whether they were the native, “as-manufactured” PP or had been retrieved. Changes in staining after sonication or sonication plus ethanol were not specific to any PP and when measured by densitometry, no significant differences were noted between cleaned PPs. Ultimately, there were no significant differences in Congo Red based on cleaning procedures, and overall intensity of staining was also similar between PPs.

### Contact angle measurements to evaluate the hydrophilic surface

**Fig. 4:**
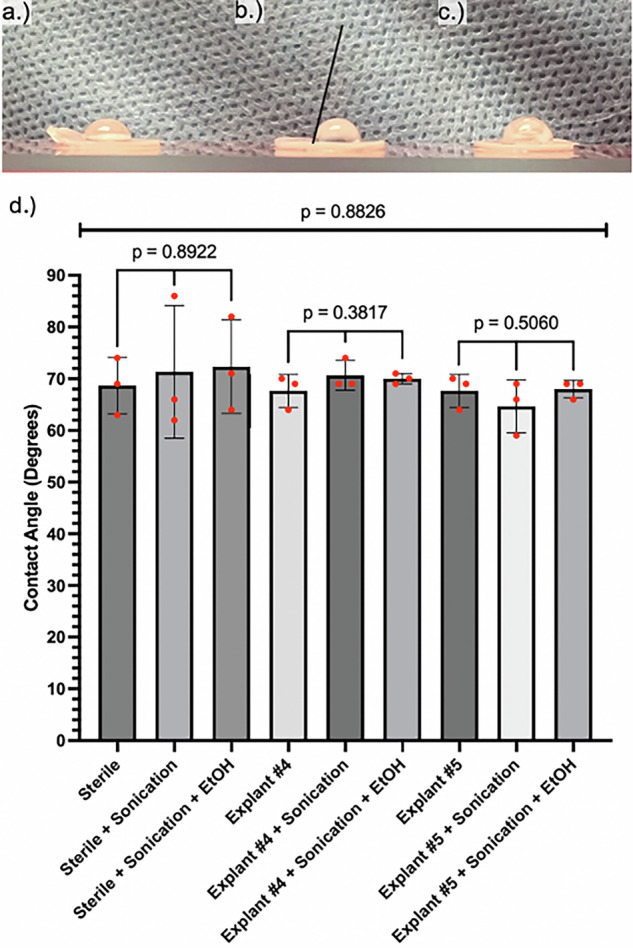
Contact Angle measurements of PP Discs. **a** Contact angle prior to sterilization; (**b**) Contact angle following sonication sterilization (line indicates tangent used to calculate contact angle); (**c**) Contact angle following sonication + EtOH sterilization. **d** Contact angle values with standard deviations for the different PPs used prior to and after sterilization.

Contact angle measurements were conducted to determine if cleaning procedures altered the surface energy of the PP surface. Native, sterile PPs showed no significant difference in contact angles between the initial surface (sterile) and after either sonication or sonication and EtOH washing followed by drying (Fig. 4). This trend was reproduced for the two explanted PPs, #4 and #5. Neither sonication nor sonication + EtOH resulted in significant differences in contact angles relative to unsterilized controls across all groups. Additionally, there existed no significant differences in contact angles between native reservoirs and explanted reservoirs, independent of sterilization status (Fig. 4).

### Antibiotic rebinding to PP hydrophilic surfaces with fluorescent labelled vancomycin

**Fig. 5:**
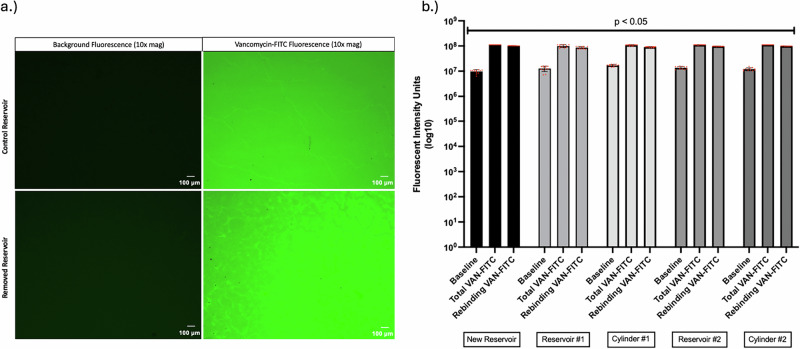
PP disc fluorescence measurements at baseline and when re-bound with FITC-labelled vancomycin (VAN-FITC). **a** Microscopic images of new and removed PPs with and without VAN-FITC. **b** Fluorescence levels from penile prosthesis (PP) surfaces re-bound with VAN-FITC. Baseline fluorescence levels of PPs re-bound with unlabeled vancomycin were gathered and subtracted from total fluorescence levels to account for potential autofluorescence of the PP material and the vancomycin moiety itself. PP surfaces were dipped in 2 mg/mL FITC-labelled or unlabeled vancomycin for 3 min, then rinsed in. NS for 5 s to remove any unbound vancomycin solution. Average net fluorescence levels with standard deviations are plotted, and for each PP group, n = 13. Statistical significance was determined by one-way ANOVA.

We assessed VAN binding to the control and explanted surfaces using fluorescently labeled (FITC) VAN. Representative control and explanted PP discs showed no background staining and these discs appeared to bind VAN-FITC avidly. In the image of the removed, explanted surface, more areas of less intense staining were apparent (Fig. 5a), although measurement of multiple fields from the different naïve and explanted PPs showed that VAN-FITC staining was similar across PPs. Upon subtracting baseline fluorescence from total fluorescence, all of the explanted PP discs had lower levels of fluorescence relative to controls (8.00 × 108 vs 7.98 × 108, p < 0.05), suggesting that VAN-FITC rebinding to these explanted surfaces was slightly reduced, albeit only within a 1% difference (Fig. 5b).

### Antiseptic materials rebound to PP hydrophilic surfaces decreases bacterial adherence

**Fig. 6:**
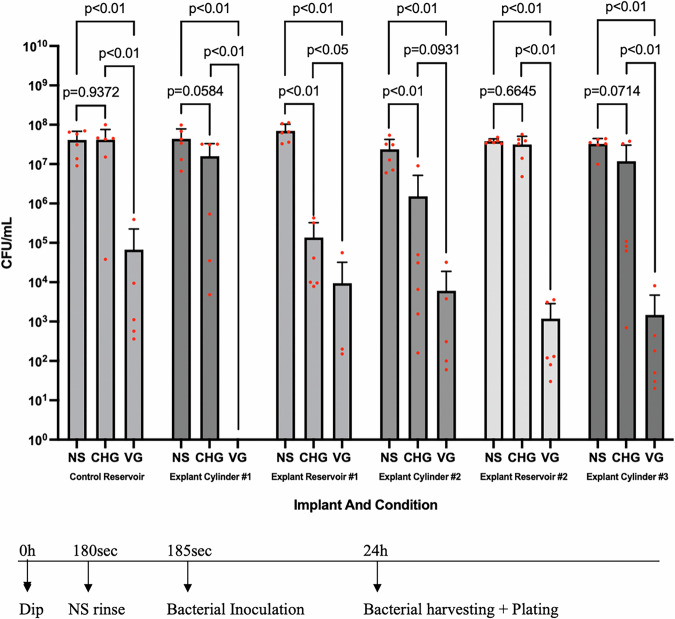
Adherent bacterial counts following antiseptic rebinding ex vivo. PP surfaces were dipped in normal saline (NS), 0.05% CHG, or 2 mg/mL vancomycin/160 μg/mL gentamicin (VG) antibiotic, and then incubated with 1 × 105 CFU/mL *S. aureus* 48 h. Recovered counts were expressed as CFU/mL (see timeline under graph). Average bacterial counts and 95% confidence intervals are plotted with n = 6 for each condition. Statistical significance was determined by Mann-Whitney U test and one-way ANOVA.

We next asked if binding either CHG or VG to the explanted surfaces would prevent bacterial colonization. All NS-rinsed surfaces showed between 10^7^–10^8^ CFU/mL of adherent bacteria. Allowing 0.05% CHG to bind to the surface and then testing for antimicrobial activity gave variable results, where no reduction in bacterial number was measured for the native surface and from 0–2.5 log (~500X) average reduction was measured on the retrieved cylinder and reservoir surfaces. Specifically, Explant Reservoir #1, and Explanted Cylinder #2 exhibited significant (~2.5 log and ~1 log decreases, respectively; p < 0.05) decreases in bacterial counts relative to NS controls after 0.05% CHG-treatment. Incubation with VG, for all surfaces, resulted in large decreases in numbers of adherent bacteria (on average 2.5–5 log decreases; explant cylinder #1 had an 8 log decrease) (Supplemental Table 1). Overall, both the control and explanted PPs in the VG groups showed significant decreases in bacterial counts relative to NS controls (p < 0.01) (Fig. 6).

## Discussion

PP revision surgery may occur for various reasons, including device malfunction, malposition, impending erosion, active erosion, and infection. Unfortunately, PP revision surgery, including single-component exchange, carries a higher infection rate than primary PP surgery [4, 6]. We asked if the hydrophilic coating can rebind antibiotics during revision surgery to offer antimicrobial protection. We report that an existing hydrophilic PP surface can rebind VG antibiotics to retard bacterial colonization and that this re-binding is more effective at retarding bacterial colonization than a similar protocol with 0.05% CHG. Overall, this VG incubation may provide beneficial antimicrobial coverage during revision surgeries when existing components are re-used (i.e., impending cylinder extrusion, reservoir herniation, or pump relocation revision). To the best of our knowledge, this is the first demonstration of the rebinding properties of the hydrophilic PP surface.

We first used Congo Red stain to evaluate the integrity of the hydrophilic coating in the operating room using an explanted PP from a patient [9]. The device stained with Congo Red demonstrated the hydrophilic surface to be intact and presumably able to rebind, similarly to the CHG-treated surfaces found by Griggs, et al. [9]. We confirmed the Congo Red staining using cleaned, sterilized (naïve and explanted PP) surfaces where we both showed the ability to rebind and the lack of effects of the cleaning protocol on this rebinding. To further test this result, we tested the contact angle, which measures wettability or hydrophilicity, on the different surfaces as a function of retrieval and of cleaning. No differences in contact angles were measured, suggesting that the retrieved PPs showed similar surface properties and ability to bind as the naïve surfaces [13]. We then quantified the degree to which rebinding occurs in explanted devices, using VAN-FITC. We found that all explanted PPs, when dipped in VAN-FITC, had fluorescence levels within 1% of that of a new PP, thereby confirming that PP hydrophilic surfaces can rebind antibiotics, albeit with marginally diminished efficacy. This pattern is observed upon microscopic examination as well; explanted PP surfaces had more areas of less intense staining relative to native controls. We finally performed microbiology studies to evaluate whether the antimicrobial rebinding properties of the hydrophilic surface conferred any antimicrobial protection. Each of the explanted PPs dipped in VG reduced bacterial counts relative to their respective NS controls, with reductions in bacterial counts comparable to that of a new PP.

Our prior studies have shown that a PP surface dipped in and irrigated with 0.05% CHG demonstrated little to no antimicrobial activity. This was in stark contrast to PP surfaces dipped in and irrigated with VG, where bacterial colonization was markedly reduced. In this study, [re]binding of 0.05% CHG to the explanted hydrophilic surfaces resulted in statistically significant antimicrobial activity in 2/5 of the explanted PP components. Only one of these PP explanted reservoirs demonstrated a ~2.5 log reduction, which is associated with good antimicrobial activity, while the other PP reservoirs only demonstrated an approximately 1 log reduction. Significant antimicrobial activity was observed in all explanted hydrophilic surfaces [re]bound with VG, with all reductions much greater than that measured with bound CHG alone [8]. Thus, independent of the known antimicrobial efficacy of 0.05% CHG or of VG, both of which have been extensively studied, our data suggest that there are differences in the ability of the VG vs. the 0.05% CHG to bind to the PP surface [16, 17]. These differences could be by virtue of molecular size, charge, or even interference by existing proteins on the PP. Further studies are needed to better elucidate the hydrophilic properties of the PP surface and the molecular interactions between the PP surface and commonly used antimicrobial solutions.

A prior study by Barham et al. showed that single-component exchange results in an over 3-fold greater infection rate over total device replacement [7]. Perhaps using an existing hydrophilic surface, when able, to rebind antibiotic solutions may reduce infection rates compared to single-component exchange surgery. Specifically, in this study, we demonstrate that hydrophilic PP surfaces rebind antibiotic solutions to confer antimicrobial activity. Recreation of the antimicrobial surface, via rebinding of antibiotics to the hydrophilic surface, may allow for device component reuse in revision surgery without compromising protection against subsequent infection. This would result both in reduction in material cost and in the patient cost and psychosocial effects of the potential for infection associated with revision surgery.

This study does not aim to ascertain which antiseptic solutions confer the greatest antimicrobial protection against the broadest spectrum of organisms. Rather, our study aims to provide the first evaluation of the rebinding and “re-elution” properties of the hydrophilic surface ex vivo. MSSA is chosen as the test organism as it remains both a historical and present-day cause of PP infection. Gross et al. demonstrated that Staphylococcal species were responsible for 34.7% of infections, of which MSSA was the 4th most common causative organism of infection and made up 10.5% of all organisms. Comparatively, *E. coli* was the most frequent organism at 18.3% [18]. Importantly, MSSA has sensitivity to vancomycin, gentamicin, and CHG making it an appropriate test organism for demonstrating the rebinding and subsequent elution of vancomycin off the explanted hydrophilic surface, using bacterial counts as our proxy [8].

It is important to note that this is an in vitro study; clinical studies are necessary to test if our in vitro findings translate into the surgical environment. Importantly, studies will be needed to better evaluate the safety and benefit of rebinding antibiotic to implanted devices and how this may reduce infection. This present study is limited by its ex vivo and in vitro design, which does not account for unique patient factors that may affect surgical outcomes, including implantation time prior to removal. In addition, the small sample size of explanted devices limits the generalizability of the study. Finally, this rebinding study was limited to studying the expandable material of the cylinders and reservoirs; it did not comprehensively study the pump, the rear-tip extenders, proximal PP, lock-out valve of the reservoir, which are all coated by the manufacturer with a hydrophilic coating. Of note, we did not observe Congo Red staining of the rear tip extenders, and the retrieved rear-tip extenders which will be studied in future experiments.

The PP’s hydrophilic surface of the reservoir and cylinders remains largely intact after implantation and may be able to be reused during revision surgery based on Congo Red staining and VAN-FITC binding studies ex vivo. Additionally, while the hydrophilic surface can rebind VG to reduce bacterial colonization, the rebinding properties of 0.05% CHG appear to be weak as evidenced by only small reductions in bacterial colonization. Further studies are needed to determine whether antimicrobial rebinding may protect against infection in clinical practice.

## Supplementary information

Supplemental Table

## Data Availability

The data that support the findings of this study are available from the corresponding author upon reasonable request.
