# Supplementary material for: Hydrophilic inflatable penile prosthesis surface coatings readily rebind antibiotics and maintain antimicrobial efficacy ex vivo
Source: Int J Impot Res. 2025 Jun 11;38(2):134–40. doi: 10.1038/s41443-025-01104-8 (PMC12893921; doi:10.1038/s41443-025-01104-8)
Supplement: Supplementary file 1 — Supplemental Table [file 41443_2025_1104_MOESM1_ESM.docx]

*Supplemental Table 1*: Recovered Bacterial Counts Following Bacterial Incubation Assay. Dipping PP discs in VG resulted in statistically significant decreases in adherent bacterial counts across all groups, relative to NS controls.

| **Control Reservoir** | | | **Explant Cylinder #1** | | | **Explant Reservoir #1** | | | **Explant Cylinder #2** | | | **Explant Reservoir #2** | | | **Explant Cylinder #3** | | |
| --- | --- | --- | --- | --- | --- | --- | --- | --- | --- | --- | --- | --- | --- | --- | --- | --- | --- |
| **(CFU/mL) (n=6)** | | | | | | | | | | | | | | | | | |
| **NS** | **CHG** | **VG** | **NS** | **CHG** | **VG** | **NS** | **CHG** | **VG** | **NS** | **CHG** | **VG** | **NS** | **CHG** | **VG** | **NS** | **CHG** | **VG** |
| 6.5 x 10^7^ | 4.3 x 10^7^ | 0 | 3.4 x 10^7^ | 5.4 x 10^5^ | 0 | 6.6 x 10^7^ | 4.3 x 10^5^ | 0 | 6.0 x 10^6^ | 160 | 0 | 4.0 x 10^7^ | 1.4 x 10^7^ | 3.6 x 10^3^ | 9.9 x 10^6^ | 8.2 x 10^4^ | 50 |
| 5.8 x 10^7^ | 4.6 x 10^7^ | 9.4 x 10^3^ | 1.3 x 10^7^ | 3.2 x 10^7^ | 0 | 1.1 x 10^8^ | 4.1 x 10^4^ | 0 | 1.3 x 10^7^ | 6.6 x 10^3^ | 3.2 x 10^5^ | 3.9 x 10^7^ | 4.2 x 10^7^ | 130 | 4.4 x 10^7^ | 3.8 x 10^7^ | 180 |
| 9.0 x 10^6^ | 1.0 x 10^8^ | 1.1 x 10^3^ | 9.8 x 10^7^ | 4.8 x 10^3^ | 0 | 3.3 x 10^7^ | 9.6 x 10^3^ | 0 | 7 x 10^6^ | 9.0 x 10^6^ | 3.8 x 10^3^ | 3.5 x 10^7^ | 4.8 x 10^6^ | 3.1 x 10^3^ | 3.5 x 10^7^ | 6.2 x 10^4^ | 20 |
| 7.0 x 10^7^ | 1.5 x 10^7^ | 570 | 6.6 x 10^6^ | 3.2 x 10^7^ | 0 | 1.1 x 10^8^ | 7.8 x 10^3^ | 5.6 x 10^4^ | 5.4 x 10^7^ | 1.6 x 10^3^ | 310 | 3.4 x 10^7^ | 5.7 x 10^7^ | 120 | 3.3 x 10^7^ | 3.3 x 10^7^ | 440 |
| 1.4 x 10^7^ | 4.5 x 10^7^ | 360 | 6.8 x 10^7^ | 3.1 x 10^7^ | 0 | 3.6 x 10^7^ | 3.2 x 10^5^ | 200 | 3.1 x 10^7^ | 3.1 x 10^4^ | 100 | 3.3 x 10^7^ | 3.9 x 10^7^ | 80 | 3.1 x 10^7^ | 700 | 8.1 x 10^3^ |
| 3.1 x 10^7^ | 3.8 x 10^4^ | 3.9 x 10^5^ | 4.3 x 10^7^ | 3.5 x 10^4^ | 0 | 6.2 x 10^7^ | 1.0 x 10^4^ | 150 | 3.2 x 10^7^ | 5.0 x 10^4^ | 60 | 4.8 x 10^7^ | 3.3 x 10^7^ | 30 | 4.2 x 10^7^ | 1.1 x 10^5^ | 30 |

NS: Normal Saline

VG: Vancomycin + Gentamicin

CHG: Chlorohexidine gluconate
